# Toll-like receptor signaling pathway involved in pathogenesis of thromboangiitis obliterans through activating of NF-κB

**DOI:** 10.1016/j.clinsp.2024.100357

**Published:** 2024-04-18

**Authors:** Facai Guo, Yan Bi, Jiangyan Yin, Yi Guo

**Affiliations:** a Department of Vascular Surgery, Lanzhou University Second Hospital, Gansu, China; b Department of Laboratory Medicine Center, Lanzhou University Second Hospital, Gansu, China; c Department of Ultrasound, The First Affiliated Hospital of Chongqing Medical University, Chongqing, China; d Department of General Surgery, Chongqing University Central Hospital (Chongqing Emergency Medical Center), Chongqing, China

**Keywords:** Thromboangiitis obliterans, TLR signaling pathway, MyD88, TRIF, NF-κB, Pathogenic mechanism

## Abstract

**Objectives:**

The pathogenic mechanisms of Thromboangiitis Obliterans (TAO) are not entirely known and autoimmune inflammation plays a vital role in the initiation and continuance of TAO activity. The authors investigated in this study the role of the TLR signaling pathway in the pathogenesis of TAO.

**Methods:**

First, the authors detected the expressions of MyD88, TRIF and NF-κB in vascular walls of 46 patients with TAO and 32 patients with trauma and osteosarcoma by western blot assay. Second, the authors detected the cellular localization of MyD88, TRIF and NF-κB in vascular walls of patients with TAO by immunofluorescent assay.

**Results:**

The protein expressions of MyD88, TRIF and NF-κB were much higher in vascular walls of TAO patients (*p* < 0.05). Higher expressions of MyD88 and NF-κB were detected both on vascular endothelial and vascular smooth muscle cells of TAO patients. However, higher expression of TRIF was just detected on vascular smooth muscle cells of TAO patients.

**Conclusions:**

These dates suggest that the TLR signaling pathway might play an important role in the pathogenesis of TAO, it might induce vasospasm, vasculitis and thrombogenesis to lead to the pathogenesis and progression of TAO.

## HIGHLIGHT

• The pathogenic mechanisms of Thromboangiitis Obliterans (TAO) are not entirely known and autoimmune inflammation plays a vital role in the initiation and continuance of TAO activity. The authors investigated in this study the role of the TLR signaling pathway in the pathogenesis of TAO.
• First, the authors detected the expressions of MyD88, TRIF and NF-κB in vascular walls of 46 patients with TAO and 32 patients with trauma and osteosarcoma by western blot assay. Second, the authors detected the cellular localization of MyD88, TRIF and NF-κB in vascular walls of patients with TAO by immunofluorescent assay.
• The protein expressions of MyD88, TRIF and NF-κB were much higher in the vascular walls of TAO patients (*p* < 0.05). Higher expressions of MyD88 and NF-κB were detected both on vascular endothelial and vascular smooth muscle cells of TAO patients. However, higher expression of TRIF was just detected on vascular smooth muscle cells of TAO patients.
• These dates suggest that the TLR signaling pathway might play an important role in the pathogenesis of TAO, it might induce vasospasm, vasculitis and thrombogenesis to lead the pathogenesis and progression of TAO.

## Background

Thromboangiitis obliterans (TAO), also known as Buerger's disease, was first described in 1908 by Buerger.^1^ It is a non-atherosclerotic inflammatory disorder of unknown etiology and can affect small and medium-sized arteries and veins in the upper and lower extremities.^2^ Although the inflammatory reactions of vasal intima have been shown in patients with TAO, the pathogenesis of TAO is still not explained exactly.^3^ Additionally, it is generally accepted that autoimmune inflammation is an ultimate pathogenic factor of TAO,^1^, ^2^, ^3^ but the action mechanism of autoimmune inflammation in patients with TAO remains unknown.

Toll-like receptor signaling pathways (TLRs) are one of the most deeply researched signaling pathways related to inflammatory diseases.^4^, ^5^, ^6^ TLRs are one kind of protein molecule involved in nonspecific immunity and it is also a bridge between nonspecific immunity and specific immunity.^7^ As public data described, TLRs can be triggered via two signaling pathways, named Myeloid Differentiation factor 88 (MyD88) dependent signaling pathway and MyD88 independent signaling pathway (also known as TRIF signaling pathway).^8^ Interestingly, both two signaling pathways may work by activating the downstream NF-κB signaling pathway.^9^ However, whether TLRs/MyD88(TRIF)/NF-κB signaling pathways are involved in the pathogenesis of TAO is still elusive.

In the present study, the authors ascertained the protein levels of MyD88, TRIF and NF-κB in the vascular walls of TAO patients and determined the subcellular localization of MyD88, TRIF and NF-κB in the vascular walls of TAO patients. These findings preliminarily uncovered that TLRs/MyD88(TRIF)/NF-κB signaling pathways are involved in the pathogenesis of TAO, which may provide more therapeutic targets for TAO patients.

## Materials and method

### Patients and sample collection

From January 2015 to December 2019, a total of 46 patients with TAO were admitted to the first affiliated hospital of Chongqing Medical University and affiliated central hospital of Chongqing University. TAO was diagnosed via color Doppler flow imaging instrument, manifesting as peripheral arterial ischemia to varying degrees. The inclusion criteria of TAO patients were: i) History of smoking; ii) Age less than 50-years old; iii) Occlusion on infrapopliteal/upper extremity artery and/or wandering phlebitis engagement. Patients with hepatorenal dysfunction, proximal limb arterial embolism, atherosclerosis, hematological system diseases and other autoimmune diseases were excluded. Additionally, 32 individuals served as the controls. Patients with hypertension, hyperlipidemia and other cardiovascular and cerebrovascular organic diseases were excluded. The clinical characteristics of the patients are reported in Table 1. The vascular tissues were obtained via surgery and stored at -80°C for succeeding experiments. The study protocols were approved by the research ethics and scientific committee of the first affiliated hospital of Chongqing Medical University and the affiliated central hospital of Chongqing University, and all subjects gave informed consent.

**Table 1:** Clinical characteristics of patients.

	TAO group	Control group
	n (%)	n (%)
Mean age ± SD	35.2 ±7.3	38.1 ± 16.4
Gender (M/F)	46/0 100/0	18/14 56.3/43.7
Mean ABI	0.267 ± 0.143	1.023 ± 1.045
Previous smoking	46 100	15 46.9
History of intermittent claudication	46 100	0 0
Pain at rest (narcotic requirement)	46 100	0 0
Ischemic nonhealing ulcer	38 82.6	0 0
Thrombophlebitis	36 78.3	0 0
Raynaud's phenomenon	35 76.1	0 0
Previous treatments with drugs		
Aspirin	44 95.7	2 6.3
Warfarin	3 6.5	0 0
Iloprost	46 100	0 0
Previous amputation		
Major/minor	3/7 6.5/15.2	0/0 0/0
Distal bypass graft		
Below knee/crural arteries	8/0 17.4/0	0/0 0/0
Sympathectomy	31 67.4	0 0
Immunosuppression	0 0	0 0
Malnutrition	10 21.7	2 6.3

### Western blot assays

Vascular tissues were lysed in RIPA buffer containing 50 mM Tris–HCl, 150 mM NaCl, 1% NP-40, 0.1% SDS, 0.5% sodium deoxycholate, 2 Mm sodium fluoride, 2 mM Na_3_VO4_2_, 1 mM EDTA, and 1 mM EGTA, and then analyzed by western blotting as previously described.^10^ In brief, samples (20 µg of total protein or 1 mg of total cell proteins) were loaded onto SDS-PAGE gels (Invitrogen) and separated by size using electrophoresis. Proteins were then transferred to PVDF membranes for 1h. The membranes were then blocked for 1h with 5% non-fat milk. Membranes were incubated with primary antibodies against MyD88 (Abcam biotechnology, inc, USA; 1:500 dilution), TRIF (Abcam; 1:500 dilution) and NF-κB (Abcam; 1:500 dilution) at 4°C overnight. After incubation with horseradish peroxidase-conjugated secondary antibody (1:1000 dilution) for 3h at 37°C. Proteins were detected by ECL chemiluminescence and analyzed by densitometry with image software.

### Immunofluorescent assay

Frozen tissues were fixed with 4% paraformaldehyde for 24h and then cut into 20-μm-thick sections. Sections were incubated by mouse anti-MyD88/TRIF/NF-κB (Abcam; 1:100 dilution), mouse anti-α-SMA (a vascular smooth muscle cell marker; Abcam; 1:250 dilution) and mouse anti-CD31 (a vascular endothelial cell marker; Abcam; 1:250 dilution) primary antibodies at 4°C overnight. Then, the slices were incubated with an anti-mouse secondary antibody (1:100 dilution) at 25°C for 60 min, followed by adding freshly prepared 0.02% diaminobenzidine for 5 min. Tissue sections were observed and photographed under a laser confocal microscope.^11^

### Statistical analysis

Statistical Package for the Social Science software (version 19.0; SPSS Concepts) was used for the data analysis. Measurement data were expressed as mean ± SD and statistically evaluated by independent-samples t-test; *p* < 0.05 was considered to indicate a statistically significant difference.

## Result

### Protein expressions of MyD88, TRIF and NF-κB in vascular tissues of two groups

**Fig. 1:**
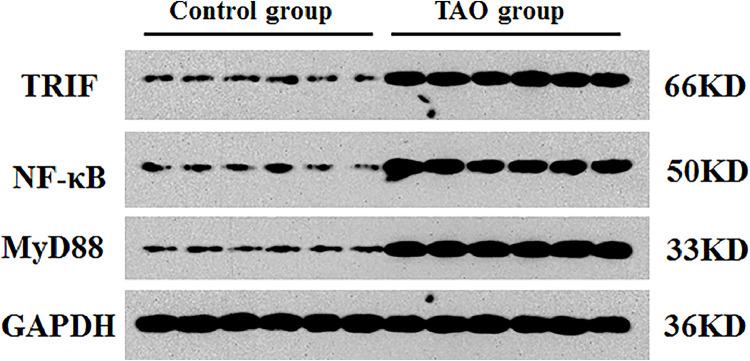
The protein levels of MyD88, TRIF and NF-κB in vascular tissues of two groups were determined via Western blotting.

The authors found that the protein levels of MyD88 (0.763 ± 0.041), TRIF (0.806 ± 0.015) and NF-κB (0.785 ± 0.032) in TAO group were dramatically elevated compared to those in the control group (MyD88 [0.188 ± 0.021], TRIF [0.162 ± 0.017] and NF-Κb [0.175 ± 0.011]) (Table 2, Fig. 1) (*p* < 0.05).

**Table 2:** The protein expression of MyD88, TRIF and NF-κB in in vascular tissues of two groups.

Group	n	MyD88	TRIF	NF-κB
Control group	46	0.188 ± 0.021	0.162 ± 0.017	0.175 ± 0.011
TAO group^a^	32	0.763 ± 0.041	0.806 ± 0.015	0.785 ± 0.032

a *p* < 0.01 vs. control group.

### Subcellular localization of MyD88 and TRIF in vascular tissues of TAO group

**Fig. 2:**
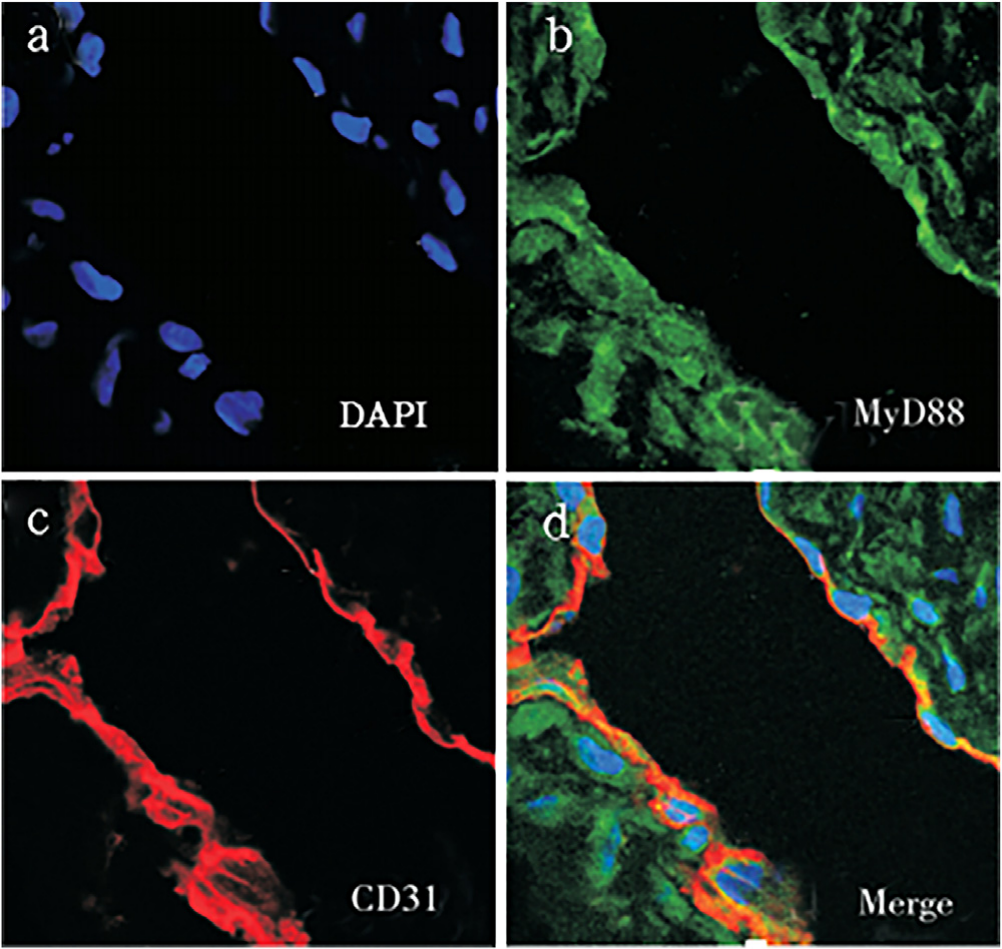
Co-expression of MyD88 with CD31 (a‒d) in the vascular endothelial cells of vascular tissues of TAO group (magnification × 400).

**Fig. 3:**
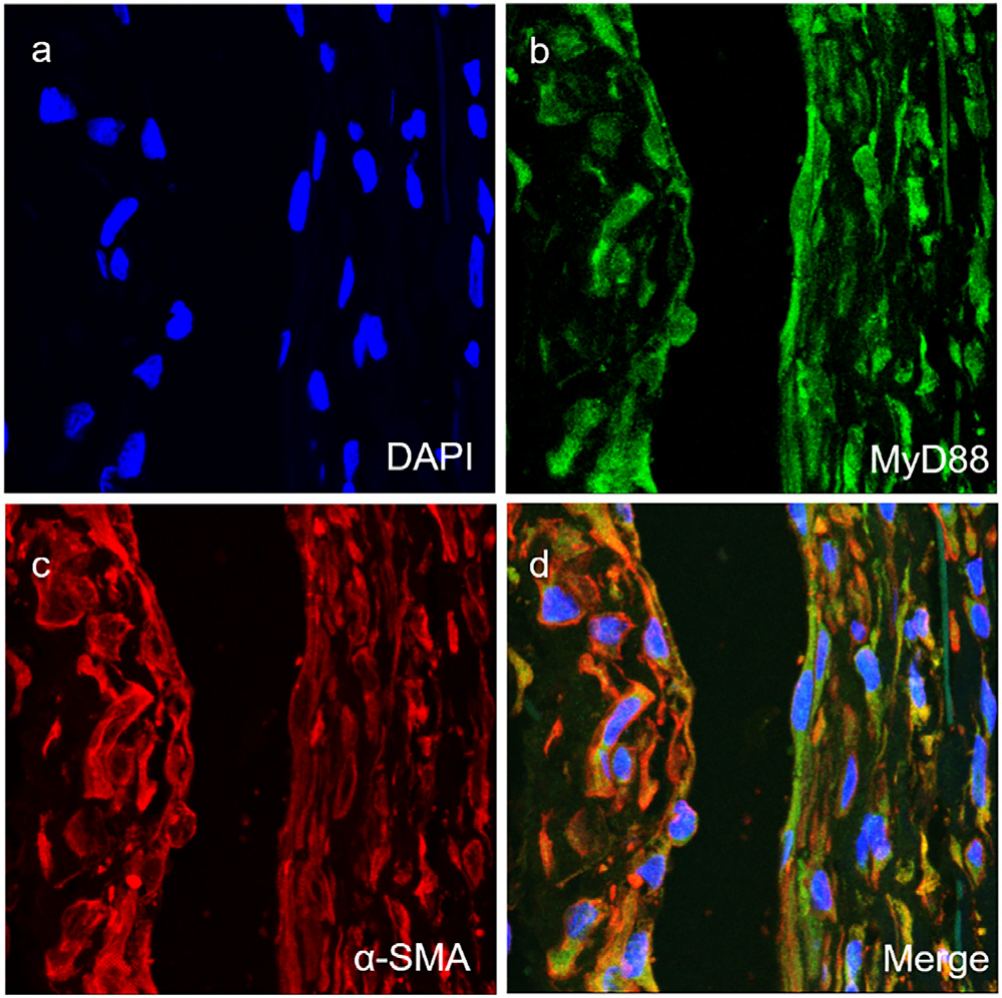
Co-expression of MyD88 with SMA (a‒d) in the vascular smooth muscle cells of vascular tissues of TAO group (magnification × 400).

**Fig. 4:**
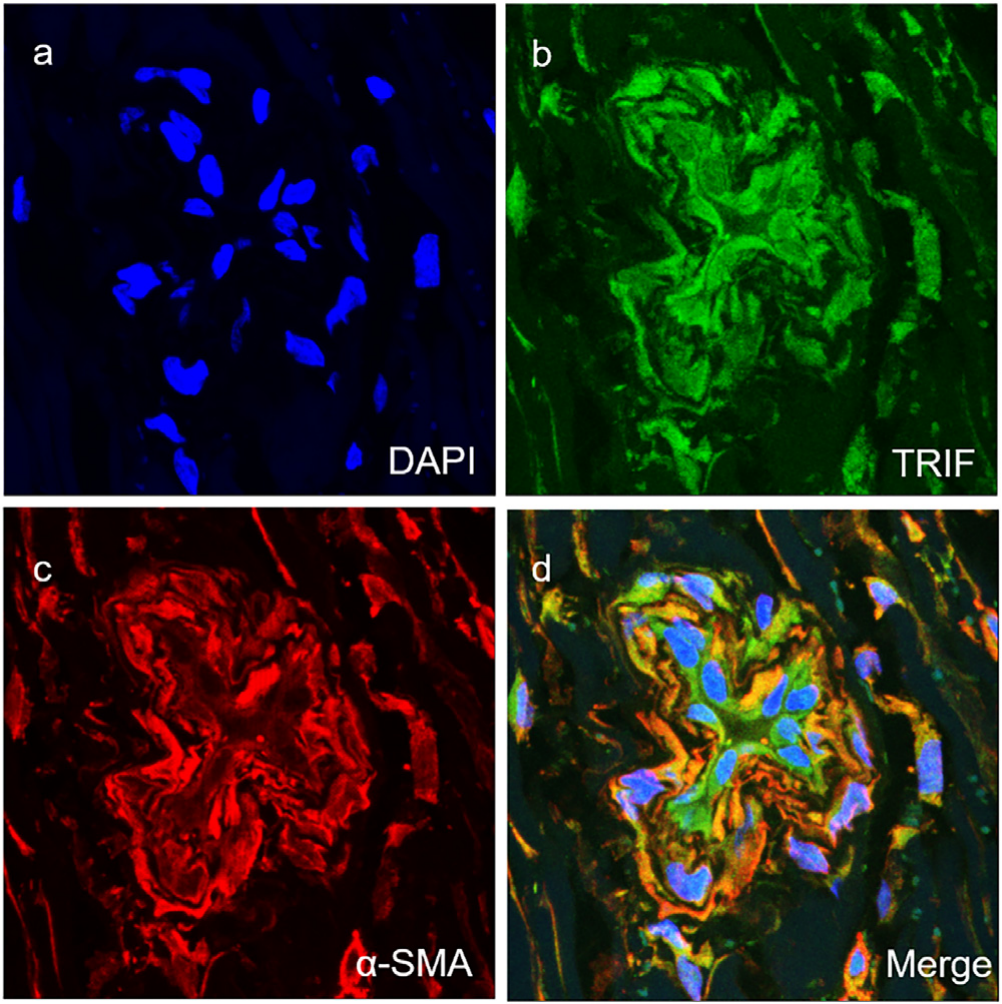
Co-expression of TRIF with SMA (a‒d) in the vascular smooth muscle cells of vascular tissues of TAO group (magnification × 400).

The subcellular localization of MyD88 and TRIF in vascular tissues of TAO patients were then ascertained. The authors found that MyD88 was mainly located in vascular endothelial cells (Fig. 2) and vascular smooth muscle cells (Fig. 3). Meanwhile, TRIF was observed to be located in vascular smooth muscle cells (Fig. 4).

### Subcellular localization of NF-κB in vascular tissues of TAO group

**Fig. 5:**
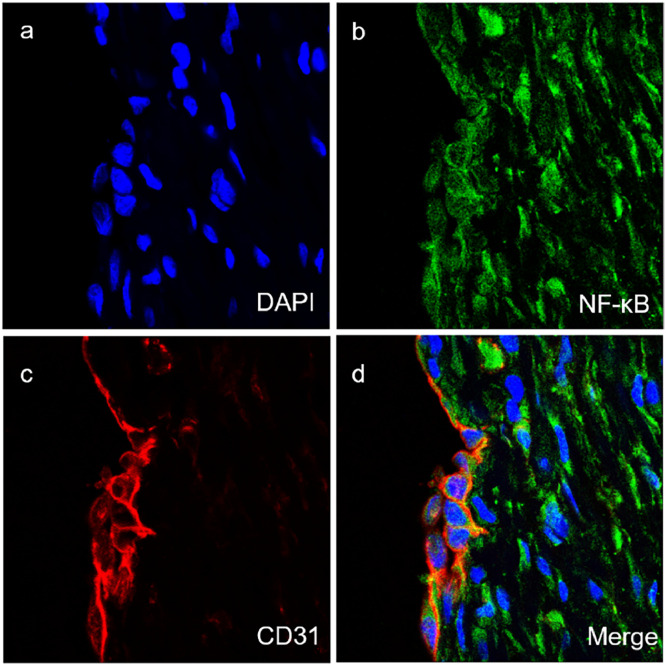
Co-expression of NF-κB with CD31 (a‒d) in the vascular endothelial cells of vascular tissues of TAO group (× 400).

**Fig. 6:**
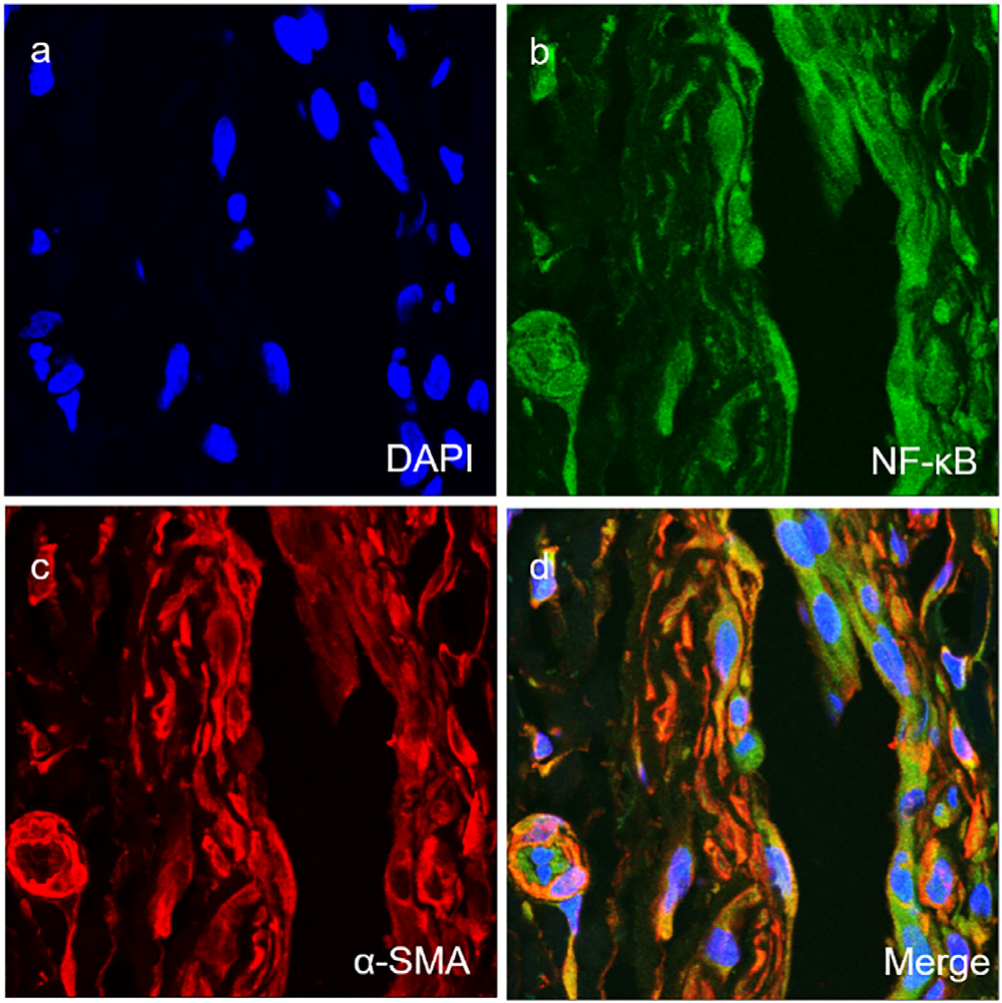
Co-expression of NF-κB with SMA (a‒d) in the vascular smooth muscle cells of vascular tissues of TAO group (× 400).

For further detection of the subcellular localization of NF-κB in vascular tissues of TAO group, the immunofluorescent assay was used. The results showed that NF-κB protein (Fig. 5-b, green) and vascular endothelial cell marker CD31 (Fig. 5-c, red) were co-expressed evidently (Fig. 5-d, yellow). Also, the results showed that NF-κB protein (Fig. 6-b, green) and vascular smooth muscle marker SMA (Fig. 6-c, red) were co-expressed evidently (Fig. 6-d, yellow). These results implied that NF-κB was also located in vascular endothelial cells and vascular smooth muscle cells.

## Discussion

More than 100 years have passed as the first description of TAO, but the pathogenesis of TAO is still unknown.^12^ Previous studies have indicated that tobacco-induced autoimmune inflammation is closely related to the pathogenesis of TAO ^13^, but the mechanisms of occurrence and development of autoimmune inflammation are still ambiguous.^14^ It is acknowledged that autoimmune inflammation is a complex molecular biological process involving multiple inflammatory pathways.^15^, ^16^, ^17^ Interestingly, public data showed that the TLRs signaling pathway, acting as a widespread innate immune pathway, can activate signaling pathways that result in immune responses and autoimmune inflammation.^18^,^19^ However, whether TLRs is involved in the onset and progression of TAO is still not determined. In the current study, the authors preliminarily detected the expression and subcellular localization of TLRs-related signaling pathways in vascular tissues of patients with TAO patients, namely MyD88, TRIF and NF-κB.

As illustrated in many research, stimulation of TLRs triggers the activation of a common MyD88-dependent signaling pathway as well as a MyD88-independent (TRIF-dependent) signaling pathway.^20^,^21^ Both of these two pathways are strongly related to innate and adaptive immune responses that are the potential predisposing factors for autoimmune inflammation.^22^,^23^ It is generally accepted that TLRs play an important role in many autoimmune inflammation diseases, such as systemic lupus erythematosus, rheumatoid arthritis, systemic sclerosis, and Sjogren's syndrome.^24^, ^25^, ^26^ TAO is an autoimmune inflammation disease with an unexplained mechanism, the authors speculated it may be also associated with TLRs.^27^ In this study, the authors found that the expression of MyD88 and TRIF was much higher in patients with TAO. Meanwhile, the results of subcellular localization showed that MyD88 was mainly located in vascular endothelial cells and vascular smooth muscle cells, while TRIF was observed to be located in vascular smooth muscle cells. According to the dates, the authors found that the main inflammatory injuries of TAO were located at vascular endothelial cells and vascular smooth muscle cells. Also, higher expressions of MyD88 and TRIF in those cells indicated that activation of TLRs signaling pathway might participate in the pathogenesis and progression of TAO.

For further determination of the relationships between TLRs and TAO, the expression of NF-κB, an important downstream transcription factor of TLRs was detected.^28^ NF-κB signaling pathway as a primary inflammatory pathway has been widely confirmed to participate in many autoimmune inflammation diseases,^29^,^30^ including TAO.^31^ Many inflammatory processes can be initiated by NF-κB signaling pathways such as angiospasm, inflammatory cell infiltration, and thrombosis.^32^, ^33^, ^34^ Interestingly, all of those inflammatory processes were considered important pathophysiological changes to be involved in the progression of TAO.^35^,^36^ In the present study, the authors found that the expression of NF-κB was much higher in patients with TAO compared to that of control individuals. At the same time, NF-κB was mainly located in the nucleus and cytoplasm of vascular endothelial cells and vascular smooth muscle cells. The results evidenced that the activation of NF-κB signaling pathway may have participated in the pathogenesis of TAO.

Some limitations have also existed in this study. First, the NF-κB signaling pathway can promote inflammatory cell infiltration by activation of intercellular adhesion molecule-1, vascular cell adhesion molecule-1, and inflammatory factors.^37^,^38^ Second, NF-κB signaling pathway may also promote thrombosis and vascular inflammation by accelerating the production of anti-neutrophil cytoplasmic antibodies, anticardiolipin antibodies, and other immune-related antibodies.^39^,^40^ The authors will elucidate these issues in the future.

## Conclusion

In a word, the current study uncovers the high expression of MyD88, TRIF and NF-κB in the vascular wall of patients with TAO, indicating that the activation of TLRs/MyD88/NF-κB and TLRs/TRIF/NF-κB signaling pathways may promote the progression of TAO by induction of vascular inflammation. These findings preliminarily evidenced that targeted therapy for the TLRs signaling pathway may be a potential therapeutic target for TAO.

## Conflicts of interest

The authors declare no conflicts of interest.
